# *Myf5* Progenitors Define the Lineage Identity of Somitic Vascular Smooth Muscle Cells

**DOI:** 10.3390/ijms27198594

**Published:** 2026-09-25

**Authors:** Donghua Hu, Kun Chen, Xifeng Chang, Xi Hu, Hongjin Chen, Xingrong Zhai, Xinkai Qu, Lei Fu, Cheng-Chao Ruan, Kailei Shi

**Affiliations:** 1 Shanghai Key Laboratory of Bioactive Small Molecules, Department of Physiology and Pathophysiology, School of Basic Medical Sciences, Fudan University, Shanghai 200032, China; 2 Department of Cardiology, Huadong Hospital, Fudan University, Shanghai 200040, China; 3 Shanghai Key Laboratory of Clinical Geriatric Medicine, Shanghai Institute of Geriatrics and Gerontology, Huadong Hospital, Fudan University, Shanghai 200040, China

**Keywords:** vascular smooth muscle cell (VSMC), *Myf5*, *MyoD*, lineage tracing

## Abstract

Vascular smooth muscle cell (VSMC) heterogeneity drives region-specific aortic pathologies, yet the roles of somite-derived lineages remain unclear. Utilizing *Myf5* and *MyoD* lineage tracing alongside a smooth muscle-specific *Myf5* conditional knockout model, we show that *Myf5*-derived cells selectively populate the thoracic aortic media, whereas the *MyoD* lineage completely spares large arteries. Early embryonic Myf5 deletion suppressed embryonic VSMC proliferation and downregulated α-SMA, reduced the synthesis of elastic, establishing its necessity in aortic morphogenesis. Upon in vitro ox-LDL challenge and calcification induction, the non-*Myf5* lineage primarily drives excessive proliferation and osteogenic transdifferentiation, whereas *Myf5* lineage VSMCs exhibit remarkable phenotypic stability and resistance to PDGF-BB-induced dedifferentiation compared to their non-*Myf5* counterparts. Collectively, our findings demonstrate a strict lineage divergence between *Myf5* and *MyoD*, suggesting that Myf5 lineage cells play an important role in maintaining aortic integrity, and uncover a lineage-imprinted plasticity that explains regional vascular heterogeneity.

## 1. Introduction

The mammalian aorta is a highly specialized conduit artery responsible for buffering the pulsatile output of the heart and maintaining systemic hemodynamic stability [1,2]. In a mature, healthy vascular system, VSMCs exhibit a quiescent, highly differentiated “contractile” phenotype characterized by the robust expression of contractile proteins, such as smooth muscle α-actin (α-SMA) and smooth muscle myosin heavy chain (SM-MHC), which collectively regulate vascular tone and compliance [3]. However, unlike terminally differentiated skeletal or cardiac muscle cells, mature VSMCs retain remarkable phenotypic plasticity. In response to local microenvironmental cues, vascular injury, or atherogenic stimuli, these cells can undergo a profound process termed “phenotypic switching” or dedifferentiation, shifting from a contractile state to a highly proliferative, migratory, and synthetic phenotype [4]. This phenotypic remodeling serves as a double-edged sword: while crucial for initial vascular repair, its chronic and uncontrolled activation constitutes a foundational driving force behind devastating cardiovascular pathologies, including atherosclerosis, restenosis, and aortic aneurysms [5].

A defining feature of vascular biology that complicates both our understanding of vessel homeostasis and the development of targeted therapies is the striking heterogeneity of the VSMC population [6]. Accumulating evidence indicates that VSMCs throughout the arterial tree exhibit distinct regional profiles regarding their baseline gene expression, secretome, proliferative capacity, and susceptibility to pathological stresses [7]. For instance, atherosclerotic plaques and aneurysms exhibit highly stereotyped topographical distributions, preferentially targeting specific segments of the aorta while leaving adjacent zones relatively spared [8,9]. This clinical and experimental phenomenon has long puzzled vascular biologists, leading to the paradigm-shifting hypothesis that the spatial heterogeneity of adult VSMCs is fundamentally pre-programmed during embryogenesis by their diverse embryonic lineages of origin [10,11].

Classical developmental studies have established that aortic VSMCs are derived from at least three distinct embryonic germ layers or progenitor populations depending on their anatomical location: the neural crest cells contribute to the aortic arch and its primary branches; the second heart field gives rise to the aortic root and ascending aorta; and the lateral plate mesoderm populates the abdominal aorta and lower-extremity vasculature [12,13,14,15]. Intriguingly, genetic fate-mapping studies have identified an additional, overlooked contributor to the vascular media: the somitic mesoderm [16,17]. During early embryonic development, somites form transiently on either side of the neural tube and segmentally differentiate into the sclerotome, dermomyotome, and myotome [18]. While traditionally recognized for giving rise to the axial skeleton, dermis, and skeletal musculature, a distinct subpopulation of progenitor cells derived from the paraxial mesoderm has been shown to migrate ventrally and contribute significantly to the tunica media of the aorta [19].

Skeletal myogenesis within the somitic lineage is strictly orchestrated by a hierarchical network of basic helix–loop–helix (bHLH) transcription factors collectively designated as myogenic regulatory factors (MRFs) [20]. Among these, Myogenic Factor 5 (Myf5) and Myoblast Determination Protein 1 (MyoD) serve as the primary upstream specification factors required for the determination and activation of skeletal muscle progenitors [21,22]. However, there is intense debate about whether these key MRFs are involved in the multilineage differentiation of somite-derived cells (especially those that eventually differentiate into the blood vessel wall). Furthermore, it remains unresolved whether all embryonic MRF-expressing populations share an equal propensity to contribute to the arterial media, or if there is a rigorous lineage divergence between *Myf5* and *MyoD* expressing progenitors during early vascular assembly.

Beyond acting as passive historical markers of embryonic origin, the direct functional requirements and cell-autonomous properties of these MRF-lineage cells within the developing and mature vasculature represent a major knowledge gap. During embryonic aortic morphogenesis, VSMC progenitor proliferation must be tightly synchronized with the synthesis and extracellular assembly of elastic laminae to withstand escalating biomechanical forces exerted by the onset of fetal circulation [23]. Disruptions in either early VSMCs expansion or the lamellar organization of elastin structures inevitably lead to structural weakness, vessel remodeling, or perinatal lethality [24]. Whether embryonic *Myf5* expression plays an active, mandatory role in driving early VSMC proliferation and directing elastic fiber assembly within the thoracic aorta—or if its transient presence is merely an evolutionary relic of somitic origin—demands urgent investigation through tissue-specific, conditional gene-ablation approaches.

Concurrently, a pivotal question in vascular medicine is how these distinct embryonic lineages dictate the cell-autonomous responses of adult VSMCs under pathophysiological stress [25]. In the context of atherosclerosis, oxidized low-density lipoprotein (oxLDL) accumulates within the subendothelial space, triggering a cascade of inflammation, oxidative stress, and lipid peroxidation [26]. This atherogenic microenvironment acts as a potent mitogen that drives VSMC hyperproliferation and phenotypic dedifferentiation, leading to the formation of foam-like or synthetic cells that populate the advanced plaque [27]. Similarly, Platelet-Derived Growth Factor-BB (PDGF-BB) is locally released by dysfunctional endothelial cells and activated platelets upon vascular injury, binding to its cognate tyrosine kinase receptors to vigorously stimulate VSMC migration and downregulate contractile proteins like SMA [28]. Given that somite-derived *Myf5 lineage* VSMCs are topographically restricted to the thoracic segment, understanding how these specific cells react to universal atherogenic stimuli (such as oxLDL and PDGF-BB) relative to their non-somite-derived neighbors is critical. Does their unique developmental history endow them with a heightened susceptibility to disease, or conversely, do they possess intrinsic molecular mechanisms that grant superior phenotypic stability and resistance against dedifferentiation pressures?

To systematically address these fundamental questions, the present study utilizes a comprehensive array of genetic lineage tracing, inducible smooth muscle-specific conditional knockout models, and primary in vitro cell-culture systems. First, by crossing *Myf5^Cre/+^* and *MyoD^Cre/+^* mice with a floxed *tdTomato* reporter, we mapped and contrasted the architectural contributions of these two primary MRF lineages across the major segments of the murine arterial tree, revealing a highly region-specific contribution exclusive to the *Myf5* lineage within the thoracic aorta, while the *MyoD* lineage completely spared the vascular wall. Second, utilizing a tamoxifen-inducible, smooth muscle-specific *Myf5* conditional knockout model, we uncovered an indispensable cell-autonomous role for *Myf5* in sustaining embryonic VSMC proliferation and ensuring the structural integrity of aortic elastic laminae during morphogenesis. Finally, utilizing primary aortic VSMCs isolated via explant outgrowth, we subjected *Myf5 lineage* cells to targeted in vitro challenges with oxLDL and PDGF-BB. Our findings demonstrate that while oxLDL stimulates the proliferation and phenotypic modulation of *Myf5*-derived cells, these cells display a remarkable, superior resistance to PDGF-BB-induced dedifferentiation compared to non-*Myf5 lineage* VSMCs. Taken together, this study provides novel insights into the somitic origin of vascular heterogeneity, establishes *Myf5* as a crucial player in aortic morphogenesis, and highlights the distinct pathophysiological plasticity of *Myf5 lineage* VSMCs, offering potential molecular targets for region-specific therapeutic interventions in vascular diseases.

## 2. Results

### 2.1. Myf5 Lineage Cells Contribute to Smooth Muscle Cells in the Thoracic Aorta

To investigate the developmental origins and topographical distribution of *Myf5 lineage* cells in the arterial system, we utilized a genetic lineage tracing approach by crossing *Myf5^Cre/+^* mice with *ROSA26R^tdTomato/+^* reporter mice (Figure 1A). Whole-mount fluorescence imaging of the isolated vasculature revealed a highly region-specific distribution of tdTomato signals. Strong tdTomato fluorescence was predominantly restricted to the thoracic aorta (TA), whereas other major arterial segments—including the carotid artery (CA), aortic arch (AA), abdominal aorta (AbA), and iliac artery (IA)—exhibited negligible or undetectable fluorescence (Figure 1B). To further characterize the cellular identity of these *Myf5*-derived cells, immunofluorescence co-staining was performed on cross-sections of various arterial segments. Consistent with the whole-mount observations, prominent tdTomato expression was exclusively captured in the tunica media of the TA, where it extensively co-localized with the VSMC marker α-SMA (Figure 1C–G). Quantitative analysis demonstrated that tdTomato-positive (td^+^) cells accounted for approximately 50% of the total SMA-positive area in the TA, contrasting sharply with the minimal presence (<5%) in the CA, AA, AbA, and FA (Figure 1H). High-magnification confocal imaging of the TA section further verified that tdTomato signals were confined to the SMA^+^ media layer and completely absent in the CD31^+^ endothelial layer (Figure 1K), demonstrating that *Myf5 lineage* cells specifically differentiate into VSMCs rather than endothelial cells in this region.

Given the substantial contribution of the *Myf5* lineage to TA smooth muscle, we next examined whether Myf5 protein continues to be expressed in adult large arteries. Western blot analysis revealed that while Myf5 protein was highly abundant in skeletal muscle (SM) used as a positive control, its expression was virtually undetectable in both adult TA and AA tissues (Figure 1I,J). Taken together, these findings indicate that a distinct subpopulation of VSMCs in the thoracic aorta originates from *Myf5*-expressing progenitor cells during development, although the expression of *Myf5* protein itself is switched off in the mature vasculature.

### 2.2. MyoD-Lineage Cells Do Not Contribute to VSMCs in Major Large Arteries

To further clarify whether other myogenic regulatory factors share a similar developmental mapping in the vasculature as *Myf5*, we performed genetic lineage tracing using *MyoD^Cre/+^*; *ROSA26R^tdTomato/+^* mice (Figure 2A). In contrast to the region-specific distribution observed in *Myf5 lineage* tracing, whole-mount fluorescence imaging of the isolated arterial tree showed a complete absence of tdTomato fluorescent signals within the primary vascular walls of all examined segments, including the carotid artery (CA), aortic arch (AA), thoracic aorta (TA), abdominal aorta (AbA), and iliac artery (IA) (Figure 2B). To confirm these macro-level observations at the cellular resolution, fluorescence histological analysis was performed on cross-sections of these distinct arterial segments. DAPI counterstaining outlined the typical ring-like structures of the vessels; however, no detectable tdTomato signal was observed within the tunica media or endothelial layers of the CA, AA, TA, or AbA (Figure 2C–F). The positive tdTomato signal observed in the surrounding skeletal muscle served as a reliable internal control, validating the efficiency of the Cre-mediated recombination system. Taken together, these results demonstrate that *MyoD*-lineage cells do not contribute to the VSMC population of major large arteries during development, establishing a distinct lineage divergence from *Myf5*.

### 2.3. Myf5 Lineage Regionally Contributes to Somitic VSMCs and Regulates Vascular Wall Homeostasis

To decipher the spatial distribution and topographic contribution of the *Myf5 lineage* to the developing vasculature, we performed genetic lineage tracing using *Myf5^Cre/+^*; *ROSA26R^tdTomato/+^* reporter embryos at embryonic day 14.5 (E14.5) (Figure 3A–C). Co-immunofluorescence staining of the tdTomato with the smooth muscle marker α-SMA revealed a highly restricted, region-specific integration pattern of *Myf5*-derivatives within the aortic media. Quantitative area analysis demonstrated that *Myf5 lineage* cells contributed substantially to the smooth muscle layer of the thoracic aorta (TA), accounting for approximately 40% of the total α-SMA area (Figure 3B,D). In contrast, the contribution of *Myf5*-derivatives dropped significantly to less than 20% in the abdominal aorta (AbA) (Figure 3C,D), and was completely negligible within the aortic arch (AA) (Figure 3A,D). These findings establish that the *Myf5 lineage* serves as a major, regionally restricted cellular source specifically for somitic vascular smooth muscle cells (VSMCs) in the thoracic segment of the aorta. We next generated a tamoxifen-inducible, smooth muscle-specific conditional knockout mouse model (*SM-MHC^CreERT2/+^*; *Myf5^Flox/Flox^*; *ROSA26R^tdTomato/+^*, CKO) and compared them with their littermate controls (*SM-MHC^CreERT2/+^*; *Myf5^Flox/+^*; *ROSA26R^tdTomato/+^*, Ctrl) (Figure S2A). Pulse-inducible activation was initiated via tamoxifen administration at E7.5–E8.5 (injection once a day), followed by tissue harvesting at E14.5 (Figure S2B). Immunofluorescence analysis revealed that in Ctrl embryos, the tdTomato^+^ cells within the tunica media exhibited robust expression of α-SMA alongside active proliferation, as evidenced by a substantial population of Ki67^+^ proliferating cells punctuated across the medial layer (Figure 3E). We observed a significant reduction in tdTomato intensity within the CKO group, implying that *Myf5* deficiency restricts the proliferation of these somite-derived smooth muscle progenitors during aortic wall development (Figure 3F). Quantification of the fluorescence signals confirmed a significant reduction in tdTomato intensity in the CKO group, indicating an impairment or loss of the targeted smooth muscle population (Figure 3G). More notably, the fluorescence intensity of α-SMA was severely diminished in CKO vessels compared to controls (Figure 3H), implying disrupted VSMC differentiation or structural destabilization of the tunica media. Furthermore, evaluation of cellular proliferation via Ki67 signal intensity revealed a profound decrease in the replication rate of medial cells within the CKO thoracic aorta (Figure 3I).

To further evaluate the impact of conditional *Myf5* deletion on the structural integrity of the embryonic thoracic aorta, histological analyses were performed on cross-sections at E14.5 using Hematoxylin and Eosin (H&E) staining and Elastic van Gieson (EVG) staining (Figure 3J,K). Quantitative measurement of the tunica media thickness revealed no statistically significant difference between Ctrl and CKO groups (Figure 3L). However, EVG staining demonstrated a significant reduction in elastic fiber thickness as well as total elastic fiber area within the aortic media of CKO embryos compared with Ctrl littermates (Figure 3M,N). These observations suggest that Myf5 deficiency may influence embryonic elastin production. To investigate the molecular mechanisms underlying these structural and cellular changes, RT-qPCR analysis was performed on thoracic aortic tissues, with β-actin (*Actb*) used as an internal control. The expression of smooth muscle contractile markers, including *Acta2* and *Myh11*, was significantly downregulated in CKO embryos (Figure 3O,P). Conversely, the expression levels of synthetic markers *Klf4* and *Spp1* showed no significant alterations between the two groups (Figure 3Q,R). In line with the reduced *Ki67* immunofluorescence staining, mRNA expression of the proliferation marker *Mki67* was markedly decreased in CKO embryos (Figure 3S), confirming a suppression of cellular proliferation upon *Myf5* deletion.

Taken together, these data indicate that *Myf5* lineage restriction is associated with localized modulation of α-SMA intensity within the *Myf5*-derived VSMC domain and contributes to the proliferation of somitic VSMCs during early aortic maturation.

### 2.4. oxLDL Promotes Proliferation and Phenotypic Switching of Primary VSMCs with Dominant Contributions from the Non-Myf5 Lineage

To further elucidate the cell-autonomous responses of *Myf5 lineage* versus non-*Myf5 lineage* vascular smooth muscle cells (VSMCs) under pro-atherogenic stress, we isolated primary VSMCs from the aortic tunica media of *Myf5^Cre/+^*; *ROSA26R^tdTomato/+^* mice using the tissue explant outgrowth method (Figure S1A). Fluorescence microscopy confirmed the successful outgrowth of td^+^ cells from the aortic explants, exhibiting the typical elongated, spindle-shaped morphology characteristic of primary VSMCs (Figure S1B).

These primary cultures were subsequently challenged with oxidized low-density lipoprotein (oxLDL) or a PBS control to evaluate lineage-specific changes in proliferative capacity and phenotypic stability. Immunofluorescence co-staining was performed using antibodies against the proliferation marker Ki67 and the contractile VSMC marker α-SMA (Figure 4A–D). Quantitative analysis revealed that oxLDL stimulation significantly accelerated the overall proliferation of these cells. Specifically, the proportion of proliferating contractile VSMCs (Ki67^+^;α-SMA^+^/α-SMA^+^) was markedly elevated in the oxLDL-treated group compared to the control (Figure 4E). Furthermore, the proliferation rate within the *Myf5 lineage* population (Ki67^+^/td^+^) also exhibited a significant increase following oxLDL exposure (Figure 4F). Concomitantly, oxLDL treatment triggered changes in α-SMA expression levels and cellular morphology across the primary cultures. Although oxLDL stimulation induced the transdifferentiation of *Myf5 lineage* smooth muscle cells ((td^+^/α-SMA^−^)/td^+^) (Figure 4G), quantitative analysis revealed a significant decrease in the overall proportion of *Myf5 lineage* cells relative to total DAPI-positive cells (td^+^/DAPI) (Figure 4H). This indicates that the non-*Myf5 lineage* (td^−^) undergoes a much more pronounced expansion and phenotypic transition, thereby diluting the relative proportion of *Myf5* derivatives within the functional VSMC pool.

TSaken together, these in vitro data demonstrate that in response to pro-atherogenic oxLDL stimulation, the non-*Myf5* lineage serves as a major contributor to the excessive proliferation and phenotypic dedifferentiation of primary VSMCs.

### 2.5. Myf5 Lineage VSMCs Exhibit Greater Resistance to PDGF-BB-Induced Phenotypic Switching

To evaluate the phenotypic stability of *Myf5 lineage* vascular smooth muscle cells (VSMCs) under migratory and proliferative stimuli, primary VSMCs isolated from *Myf5^Cre/+^*; *ROSA26R^tdTomato/+^* mice were challenged with Platelet-Derived Growth Factor-BB (PDGF-BB), a potent inducer of VSMC phenotypic modulation, or PBS control (Figure 5A–D). Under baseline conditions (PBS), the vast majority of both total cells (SMA^+^/DAPI^+^) and *Myf5 lineage* cells (α-SMA^+^/td^+^) relative resistance to PDGF-BB-induced suppression of contractile markers, with smooth muscle α (α-SMA) positivity exceeding 90% (Figure 5E). Upon PDGF-BB stimulation, a profound downregulation of SMA expression was observed across the culture, indicating successful induction of phenotypic switching. However, quantitative analysis revealed a striking divergence between *Myf5 lineage* and non-lineage cells. The percentage of total α-SMA-positive cells (α-SMA^+^/DAPI^+^) dropped, whereas the α-SMA-positive ratio within the *Myf5 lineage* population (α-SMA^+^/td^+^) remained significantly higher (Figure 5E).

Consistently, when evaluating the dedifferentiated population (α-SMA^−^), PDGF-BB treatment markedly increased the proportion of modulated cells in both sectors (Figure 5F). Crucially, the percentage of dedifferentiated cells within the *Myf5* lineage (α-SMA^−^;td^+^)/td^+^ was significantly lower than the dedifferentiation rate observed in the non-*Myf5* lineage fraction (α-SMA^−^;td^−^)/(DAPI^+^;td^−^), (*p* < 0.0001; Figure 5F). Taken together, these findings demonstrate that while PDGF-BB drives more than VSMC phenotypic modification, *Myf5 lineage* VSMCs possess a distinct cell-autonomous property that grants enhanced resistance to dedifferentiation stimuli compared to their non-*Myf5 lineage* counterparts.

### 2.6. Myf5 Lineage VSMCs Display Attenuated Calcification Capacity In Vitro

To assess whether embryonic lineage origin dictates the susceptibility of VSMCs to pro-calcifying cues, primary VSMCs isolated from *Myf5^Cre/+^*; *ROSA26R^tdTomato/+^* mice were subjected to osteogenic induction (10 mM β-glycerophosphate, 50 µg/mL L-ascorbic acid, and 10 nM dexamethasone) for 7 days (Figure 6). Alizarin Red S staining combined with endogenous tdTomato fluorescence revealed extensive formation of calcified extracellular matrix nodules across the culture (Figure 6A). Intriguingly, spatial distribution analysis demonstrated that dense calcium deposits were predominantly associated with tdTomato-negative (non-*Myf5* lineage) regions, whereas tdTomato-positive (*Myf5*-derived) cell clusters exhibited minimal nodule accumulation (Figure 6(Aa,Ab)). To further evaluate lineage-specific osteogenic transdifferentiation at the cellular level, immunofluorescence co-staining was performed for the osteogenic marker Osteopontin (OPN) and the contractile marker α-SMA (Figure 6B). While non-*Myf5* lineage cells (tdTomato^−^) robustly upregulated OPN expression upon calcification induction, *Myf5 lineage* VSMCs (tdTomato^+^) retained substantial α-SMA expression and displayed negligible OPN signal (Figure 6(B5,B6)). Line scan fluorescence intensity profiling along the cellular boundaries confirmed an inverse relationship: peak OPN fluorescence signals strictly localized to areas adjacent to, but excluded from, regions of high tdTomato intensity (Figure 6C). Together, these results demonstrate that *Myf5 lineage* VSMCs possess intrinsic resistance against osteogenic phenotypic switching and vascular calcification compared to non-*Myf5* lineage cells.

### 2.7. mRNA Expression of Phenotypic Transition Markers in Myf5 Lineage Versus Non-Myf5 Lineage VSMCs Under ox-LDL

To directly validate whether lineage origin dictates distinct cell-autonomous responses to atherogenic stress without confounding paracrine interactions, primary VSMCs from *Myf5^Cre/+^*; *ROSA26R^tdTomato/+^* mice were isolated and separated into *Myf5^+^* (PE^+^) and *Myf5^−^* (PE^−^) subpopulations via fluorescence-activated cell sorting (FACS) (Figure 7A). Sorted subpopulations were treated with ox-LDL (50 µg/mL) or PBS control for 48 h, and the mRNA expression levels of key contractile, synthetic, and proliferative markers were quantitatively analyzed by RT-qPCR (Figure 7B–F). Under baseline conditions (PBS control), *Myf5^+^* VSMCs displayed significantly higher baseline mRNA expression of contractile markers (*Acta2* and *Myh11*) compared to *Myf5^−^* VSMCs (Figure 7B,C). Upon ox-LDL stimulation, both sub-populations exhibited marked downregulation of contractile genes; however, *Myf5^+^* cells maintained higher residual levels of *Acta2* than *Myf5^−^* cells (Figure 7B). Conversely, ox-LDL challenge vigorously induced the expression of synthetic markers (*Klf4* and *Spp1*) as well as the proliferation marker (*Mki67*) (Figure 7D–F). Crucially, *Myf5^−^* VSMCs exhibited a strikingly higher induction of *Spp1* (Osteopontin) and *Mki67* compared to *Myf5^+^* VSMCs (Figure 7E,F), whereas *Klf4* upregulation was comparable between the two subsets (Figure 7D). These results collectively suggest that the non-*Myf5* lineage accounts for the predominant synthetic and proliferative transcriptional response to ox-LDL, whereas the *Myf5* lineage maintains relative transcriptomic stability.

## 3. Discussion

This study utilizes genetic lineage tracing, conditional gene knockout, and in vitro pathological stimulation models to provide insights into the roles of somite-derived myogenic regulatory factors (MRFs) in vascular heterogeneity and phenotypic plasticity. The spatial heterogeneity of VSMCs across the mammalian arterial tree represents a foundational paradigm that explains the topographical selectivity of major aortic diseases [29,30,31]. Although classical studies have comprehensively mapped the contributions of the neural crest, second heart field, and lateral plate mesoderm to the aortic wall, the exact role and cell-autonomous properties of paraxial or somitic mesoderm-derived VSMCs have remained obscure [10,11,32].

Our experimental findings explicitly demonstrate that *Myf5* and *MyoD*, despite sharing roles as master regulators of skeletal myogenesis [33], exhibit a profound developmental divergence within the vascular system. The Myf5 lineage specifically contributes to more than half of the smooth muscle population in the thoracic aortic media, whereas the MyoD lineage completely spares the vascular walls of all examined large arteries. This striking divergence indicates that the somitic progenitor pool undergoes precise lineage segregation during early embryogenesis, allowing a distinct subset of *Myf5*-expressing cells to bypass the terminal skeletal muscle program and adopt a smooth muscle fate. *Myf5* is a key transcription factor driving somitic mesoderm lineage commitment toward skeletal muscle. Its distinct silencing in mature vascular smooth muscle cells (VSMCs) reflects a tightly controlled cell fate selection mechanism. We speculate that as somite-derived progenitors transition into the VSMC fate, the activation of the SRF/Myocardin complex robustly drives contractile VSMC gene expression (e.g., α-SMA, SM22α, and SM-MHC) while competitively repressing bHLH myogenic programs including Myf5 [34,35].

Furthermore, the lack of detectable Myf5 protein expression in adult mouse arteries suggests that this factor functions predominantly as a developmentally restricted regulator during early vascular development rather than a continuous maintenance factor. From a functional perspective, *Myf5* serves as an indispensable cell-autonomous regulator of aortic structural maturation rather than a passive lineage marker. Smooth muscle-specific deletion of *Myf5* significantly suppresses embryonic VSMC proliferation at E14.5. Histological evaluation further indicated that while conditional Myf5 deficiency did not significantly alter overall aortic tunica media thickness, it was accompanied by a reduction in elastic fiber thickness. During early aortic morphogenesis, the orderly expansion of VSMC progenitors must be strictly synchronized with the extracellular assembly of elastic fibers to withstand the escalating biomechanical forces exerted by the onset of fetal circulation [36]. In vitro stimulation assays reveal that under pro-atherogenic oxLDL challenge, the non-*Myf5* lineage serves as the primary contributor to excessive cell proliferation and phenotypic dedifferentiation, highlighting a lineage-specific susceptibility to pathological vascular remodeling. Under potent PDGF-BB-induced stimulation, *Myf5 lineage* VSMCs maintain significantly higher smooth muscle α-actin (α-SMA) expression and exhibit a drastically lower dedifferentiation rate compared to their non-*Myf5* lineage counterparts. Beyond PDGF-BB-induced dedifferentiation, our in vitro calcification and FACS-sorted assays revealed potential lineage-associated differences in response to osteogenic and atherogenic stimuli. Under pro-calcifying conditions, *Myf5*-derived VSMCs showed relatively modest calcium nodule deposition and lower Osteopontin (OPN) expression compared to non-*Myf5* lineage cells. Furthermore, FACS-sorted subpopulations pointed to differential responses to ox-LDL challenge between the two groups. While ox-LDL treatment tended to reduce contractile marker levels in both subsets, the non-*Myf5* (*Myf5*^−^) population exhibited a notably stronger induction of synthetic (*Spp1*) and proliferative (*Mki67*) markers. This divergence—where non-*Myf5* VSMCs appear more prone to hyperproliferation and osteogenic transdifferentiation, while *Myf5*^+^ VSMCs tend to maintain relative phenotypic stability—might offer a complementary developmental perspective on regional variation in vascular disease susceptibility. This lineage-specific resistance trait suggests potential intrinsic molecular buffering systems within Myf5-derived cells, which may provide insights into why different aortic segments—such as the thoracic versus abdominal aorta—exhibit distinct regional susceptibility to cardiovascular pathologies [37].

Despite these insights, several limitations remain to be addressed in future investigations. First, the precise downstream transcriptional networks or epigenetic modifications permanently locked in by *Myf5* expression require further characterization via high-resolution single-cell multi-omics. In our follow-up studies, we plan to scale up primary isolation, employ fluorescence-activated cell sorting (FACS) to isolate lineage-marked populations, and perform high-throughput RNA sequencing (RNA-seq) alongside multi-marker validation to thoroughly map their transcriptomic and phenotypic profiles. Second, future studies should employ adult-inducible dual-recombinase systems to track and manipulate the *Myf5* lineage within complex in vivo disease models, such as atherosclerosis or aneurysm models. This will be critical to determine whether the lineage-specific resistance to dedifferentiation observed in vitro translates into enhanced fibrous cap stability or restricted plaque progression in vivo. We fully acknowledge that our current findings are predominantly based on in vitro cell culture systems and developmental baseline observations. While these data demonstrate a novel, lineage-restricted pattern of α-SMA expression and morphological plasticity, their direct extrapolation to complex vascular pathology remains speculative. Future studies utilizing dedicated in vivo disease models, such as atherosclerosis (ApoE^−/−^), will be essential to evaluate whether this lineage-specific plasticity functionally contributes to pathological vascular remodeling. In conclusion, this work expands our understanding of the developmental origins of vascular diversity and provides a promising framework for designing region-tailored therapeutic strategies in vascular medicine.

## 4. Materials and Methods

### 4.1. Animal Models

All animal procedures were approved by the Animal Management and Use Committee of the School of Basic Medicine, Fudan University, and performed in accordance with the institutional guidelines of Shanghai Medical College, Fudan University. The animals were housed in a specific pathogen-free facility at the Laboratory Animal Service Center of Fudan University. The housing environment was maintained on a 12 h light/dark cycle with ad libitum access to standard chow and water. *Myf5^Cre/+^* mice were sourced from Jackson Laboratory (JAX: 025524). *ROSA26R^tdTomato/+^* mice were obtained from Jackson Laboratory (JAX: 007914). *SM-MHC^CreERT2/+^* mice were obtained from Jackson Laboratory (JAX: 019079). *MyoD^Cre/+^* mice were obtained from Jackson Laboratory (JAX: 014140). *Myf5^flox/flox^* mice sourced from GemPharmatech company (T011035).

### 4.2. Whole-Mount Vascular Fluorescence Imaging

Mice were euthanized and perfused transcardially with ice-cold phosphate-buffered saline (PBS). The entire arterial tree, including the carotid artery (CA), aortic arch (AA), thoracic aorta (TA), abdominal aorta (AbA), and iliac artery (IA), was carefully dissected under a stereomicroscope. Whole-mount brightfield and endogenous tdTomato fluorescent images of the intact vasculature were captured using a fluorescence stereomicroscope (Leica DM205) to evaluate the macroscopic topographical distribution of lineage-traced cells.

### 4.3. Tissue and Cell Immunofluorescence Staining

Tissue Immunofluorescence: For cryosections, samples were embedded in OCT compound, sectioned at 10 μm, and permeabilized with 0.1% Triton X-100. Sections were blocked with 5% bovine serum albumin (BSA) or normal goat serum for 1 h at room temperature, followed by overnight incubation at 4 °C with primary antibodies: anti-α-SMA (Proteintech, 14395-1-AP), anti-Ki67 (Abcam, ab15580), and anti-CD31 (R&D, AF3628), anti-PDGFRα (R&D, MAB3221), anti-OPN (R&D, AF808). After washing with PBS, sections were incubated with corresponding Alexa Fluor-conjugated secondary antibodies (488, 594, or 647) for 1 h at room temperature. Nuclei were counterstained with DAPI. Cell Immunofluorescence: Primary VSMCs cultured on coverslips were fixed with 4% PFA for 10 min, permeabilized with 0.1% Triton X-100 for 10 min, blocked with 5% BSA, and stained following the same protocol described above. Images were acquired using a confocal laser microscope (Leica SP8).

### 4.4. Isolation, Culture, and Purity Identification of Primary Aortic VSMCs

Primary vascular smooth muscle cells (VSMCs) were isolated from the aortic tunica media of *Myf5^Cre/+^*; *ROSA26R^tdTomato/+^* mice using the tissue explant outgrowth method based on a previously described protocol [38]. Briefly, the aorta was harvested under sterile conditions, and the adventitia and endothelial layers were carefully peeled off using fine forceps. The remaining tunica media were minced into small pieces (approximately 1 mm^2^), placed onto gelatin-coated cell culture dishes, and maintained in complete culture medium for mouse aortic smooth muscle cells (Procell, CM-M076), and smooth muscle growth supplements at 37 °C in a humidified incubator with 5% CO_2_. Primary VSMCs migrated from the tissue block 5-7 days before the first tissue removal and collection. Primary VSMCs between passages 2 and 5 were used for subsequent experiments. To verify cell purity, immunofluorescence staining for the smooth muscle-specific marker alpha-smooth muscle actin (α-SMA) was performed. Over 95% of the isolated cells were confirmed as α-SMA-positive (α-SMA^+^/DAPI^+^; Figure 5E), validating the high purity of the VSMC preparations for downstream experiments.

### 4.5. In Vitro Stimulation Assays (oxLDL and PDGF-BB)

To simulate pathophysiological and pro-atherogenic stress in vitro, primary VSMCs were seeded into 6-well plates (1 × 10^5^ cells/well) and allowed to reach 60–70% confluence. Prior to treatment, cells were serum-starved in DMEM containing 0.5% FBS for 12 h to synchronize the cell cycle. oxLDL (Yiyuanbiotech, YB-002) Challenge: Cells were incubated with oxidized low-density lipoprotein (oxLDL, 50 µg/mL) or an equal volume of PBS control for 48 h. PDGF-BB (MedChemExpress, HY-P7278) Challenge: Cells were stimulated with recombinant human Platelet-Derived Growth Factor-BB (PDGF-BB, 20 ng/mL) or PBS control for 48 h to evaluate phenotypic switching kinetics and lineage resistance traits.

### 4.6. In Vitro VSMC Calcification Model

Primary vascular smooth muscle cells (VSMCs) were isolated from *Myf5^Cre/+^*; *ROSA26R^tdTomato/+^* mice using the aortic explant outgrowth method described above (4.4). After 5–7 days of initial cell migration from the tissue explants, osteogenic transdifferentiation and mineralization were directly induced. The complete culture medium was replaced with pro-calcifying medium consisting of basal medium supplemented with 10 mM β-glycerophosphate, 50 µg/mL L-ascorbic acid, and 10 nM dexamethasone [39]. Cells were incubated in the calcification-inducing medium for 7 days at 37 °C in a humidified atmosphere with 5% CO_2_, with the medium refreshed every 48 h. Following the 7-day treatment, cells were harvested or fixed for downstream analyses, including Alizarin Red S staining and immunofluorescence microscopy.

### 4.7. Alizarin Red S Staining

To evaluate calcium deposition and mineralized matrix formation, Alizarin Red S staining was performed following the 7-day calcification induction. Cells cultured in 6-well plates or on coverslips were washed twice with phosphate-buffered saline (PBS) and fixed with 4% paraformaldehyde (PFA) for 10 min at room temperature. After washing three times with distilled water (dH_2_O) to remove residual fixative, cells were incubated with 1.5% Alizarin Red S solution (Servicebio, G1038-100ML) for 20–30 min at room temperature with gentle shaking, protected from light. Non-specifically bound dye was removed by thoroughly rinsing the cells with dH_2_O four to five times until the rinse solution remained clear. Calcium deposits were visualized and photographed under a brightfield microscope combined with fluorescence microscopy (for detection of endogenous tdTomato expression).

### 4.8. Histological Analysis (H&E and EVG Staining)

Embryonic aortic tissues harvested at E14.5 were fixed in 4% paraformaldehyde (PFA), dehydrated through a graded ethanol series, embedded in paraffin, and sectioned into 5 μm thick cross-sections. Prior to staining, sections were deparaffinized in xylene and rehydrated through a descending alcohol series to water. Hematoxylin and Eosin (H&E) Staining kit (Servicebio, G1005): Rehydrated sections were stained with hematoxylin solution for 5 min, differentiated in 1% acid alcohol, and counterstained with eosin Y for 1 min. Sections were subsequently dehydrated through graded ethanol, cleared in xylene, and mounted with synthetic resin to evaluate general aortic morphometry. Elastic van Gieson (EVG) Staining: To visualize elastic laminae, sections were stained using an EVG staining kit (Solarbio, G1591) according to the manufacturer’s instructions. Briefly, sections were incubated in Verhoeff’s elastic stain working solution for 20 min, differentiated in 2% ferric chloride solution until elastic fibers were clearly delineated, rinsed in 95% ethanol, and counterstained with van Gieson’s picro-fuchsin solution for 1 min.

### 4.9. Fluorescence-Activated Cell Sorting (FACS)

Primary vascular smooth muscle cells (VSMCs) isolated from *Myf5^Cre/+^*; *ROSA26R^tdTomato/+^* mice were harvested, washed with ice-cold phosphate-buffered saline (PBS), and resuspended in sorting buffer (PBS supplemented with 2% fetal bovine serum). To discriminate viable cells, Live/Dead Fixable Near-IR Dead Cell Stain (Biolegend, Cat # 423105) was used. Cell sorting was performed on a high-speed flow cytometer (BD FACSAria III). Cells were sequentially gated to isolate the main cell population (FSC-A vs. SSC-A; P1), single cells (FSC-A vs. FSC-H; P2), and viable cells (Live-APC-Cy^−^). Based on endogenous tdTomato fluorescence intensity (PE channel), cells were separated into *Myf5^+^* (PE^+^) and *Myf5^−^* (PE^−^) subpopulations. Sorted cells were collected into complete medium for downstream culture assays.

### 4.10. Total RNA Extraction and RT-qPCR

Total RNA was extracted from VSMCs and Vascular tissue using the RNA Extraction Kit (Servicebio, Cat# G3689) following the manufacturer’s instructions, including on-column DNase I treatment. cDNA was synthesized using a reverse transcription kit (Servicebio, Cat# G3333-50). Quantitative PCR was performed using 2X Universal SYBR Green Fast qPCR Mix (ABclonal, Cat# RK21203) with the following cycling conditions: 95 °C for 3 min, followed by 40 cycles of 95 °C for 5 s and 60 °C for 30 s. Relative mRNA expression was calculated using the 2^−ΔΔCT^ method with Actb (β-actin) as the internal control. Primer sequences are listed in Table S1.

### 4.11. Protein Extraction and Western Blot Analysis

Total protein was extracted from adult mouse skeletal muscle (positive control), thoracic aorta (TA), and aortic arch (AA) tissues using RIPA lysis buffer supplemented with a protease and phosphatase inhibitor cocktail. Protein concentrations were determined using a BCA protein assay kit. Equal amounts of protein (20–30 µg per lane) were separated via 10% SDS-PAGE gels and transferred onto polyvinylidene difluoride (PVDF) membranes. Membranes were blocked with 5% non-fat milk in Tris-buffered saline with 0.1% Tween-20 (TBST) for 1 h, then incubated overnight at 4 °C with primary antibodies against Myf5 (Abclonal, A16227) and β-actin (Servicebio, GB15001). After washing with TBST, membranes were incubated with horseradish peroxidase (HRP)-conjugated secondary antibodies for 1 h. Protein bands were visualized using an enhanced chemiluminescence (ECL) detection system and quantified via densitometric analysis using ImageJ.

### 4.12. Statistical Analysis

Data are expressed as mean ± SEM. Statistical significance (*p* < 0.05) was evaluated using an unpaired Student’s *t*-test for two groups, or a one-way or two-way ANOVA followed by Tukey’s post hoc test for multiple comparisons. Specific statistical methods are indicated in the figure legends. Statistical analyses were performed using GraphPad Prism software.

## Figures and Tables

**Figure 1:**
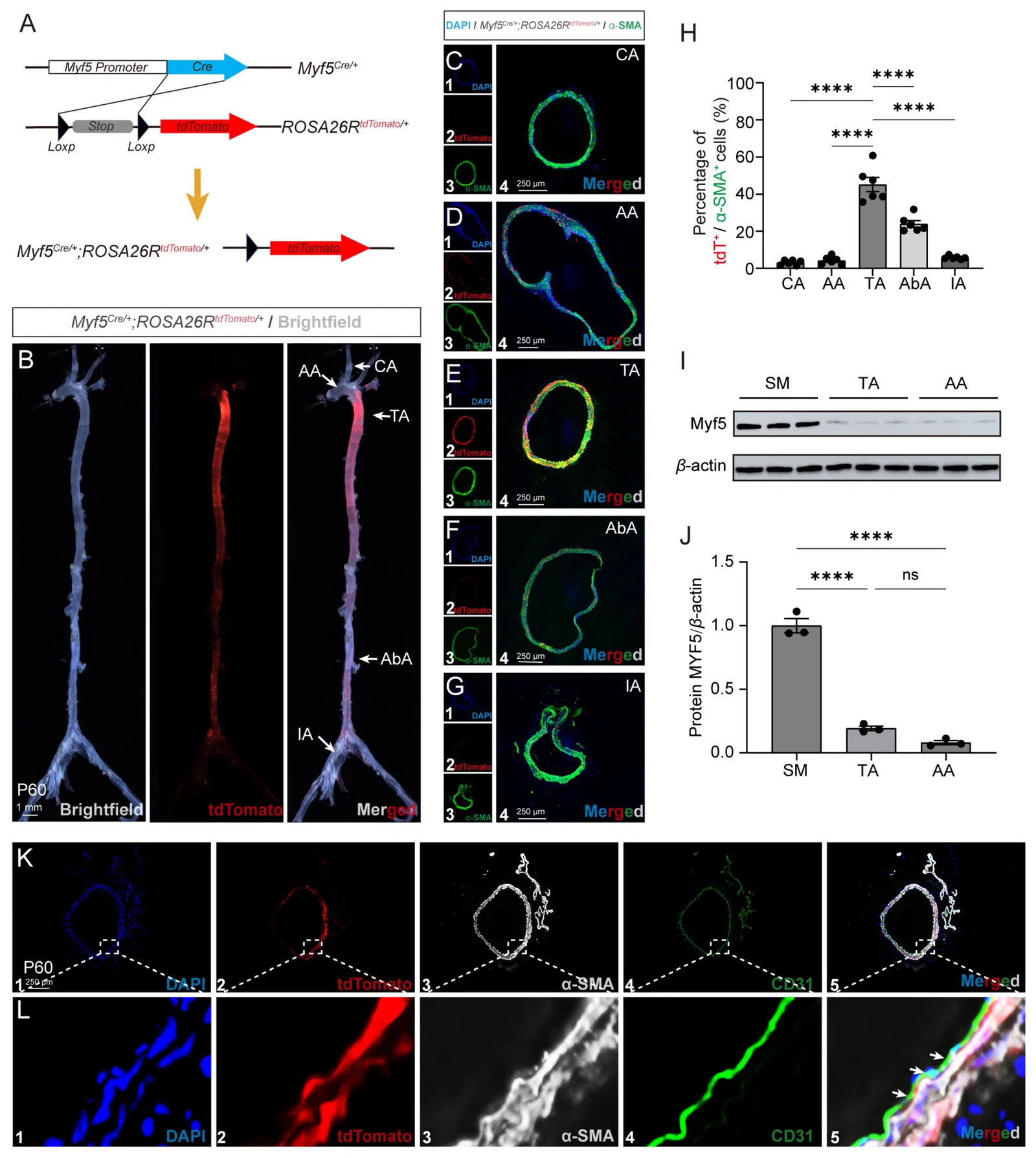
Genetic lineage tracing reveals a region-specific contribution of Myf5 lineage cells to thoracic aortic smooth muscle. (**A**) Schematic illustration of the genetic strategy used for lineage tracing of *Myf5*-derived cells using *Myf5^Cre/+^*; *ROSA26R^tdTomato/+^* mice. (**B**) Representative whole-mount brightfield, tdTomato fluorescence, and merged images of the murine aorta and its main branching arteries, including the carotid artery (CA), aortic arch (AA), thoracic aorta (TA), abdominal aorta (AbA), and iliac artery (IA). Scale bars, 1 mm. (**C**–**G**) Representative immunofluorescence images of arterial cross-sections from CA (**C**), AA (**D**), TA (**E**), AbA (**F**), and IA (**G**) stained with DAPI (blue, 1), tdTomato (red, 2), and SMA (green, 3); panel 4 shows the merged images. Scale bars: 250 µm. (**H**) Quantitative analysis of the percentage of (td^+^/α-SMA^+^) cells across different arterial segments (n = 6 per group). (**I**,**J**) Western blot analysis (**I**) and corresponding quantification (**J**) of Myf5 protein expression in adult skeletal muscle (SM), TA, and AA (n = 3 per group). (**K**) TA cross-section showing the localization of DAPI (blue, 1), tdTomato (red, 2), SMA (white, 3), and CD31 (green, 4); panel 5 shows the merged image. (**L**) Magnified views of the white dashed boxed areas in panel (**K**) (**K1**–**K5**). Scale bars: 250 µm. Data are presented as means ± SEM. **** *p* < 0.0001. ns = not significant. One-way ANOVA was used for (**H**,**J**).

**Figure 2:**
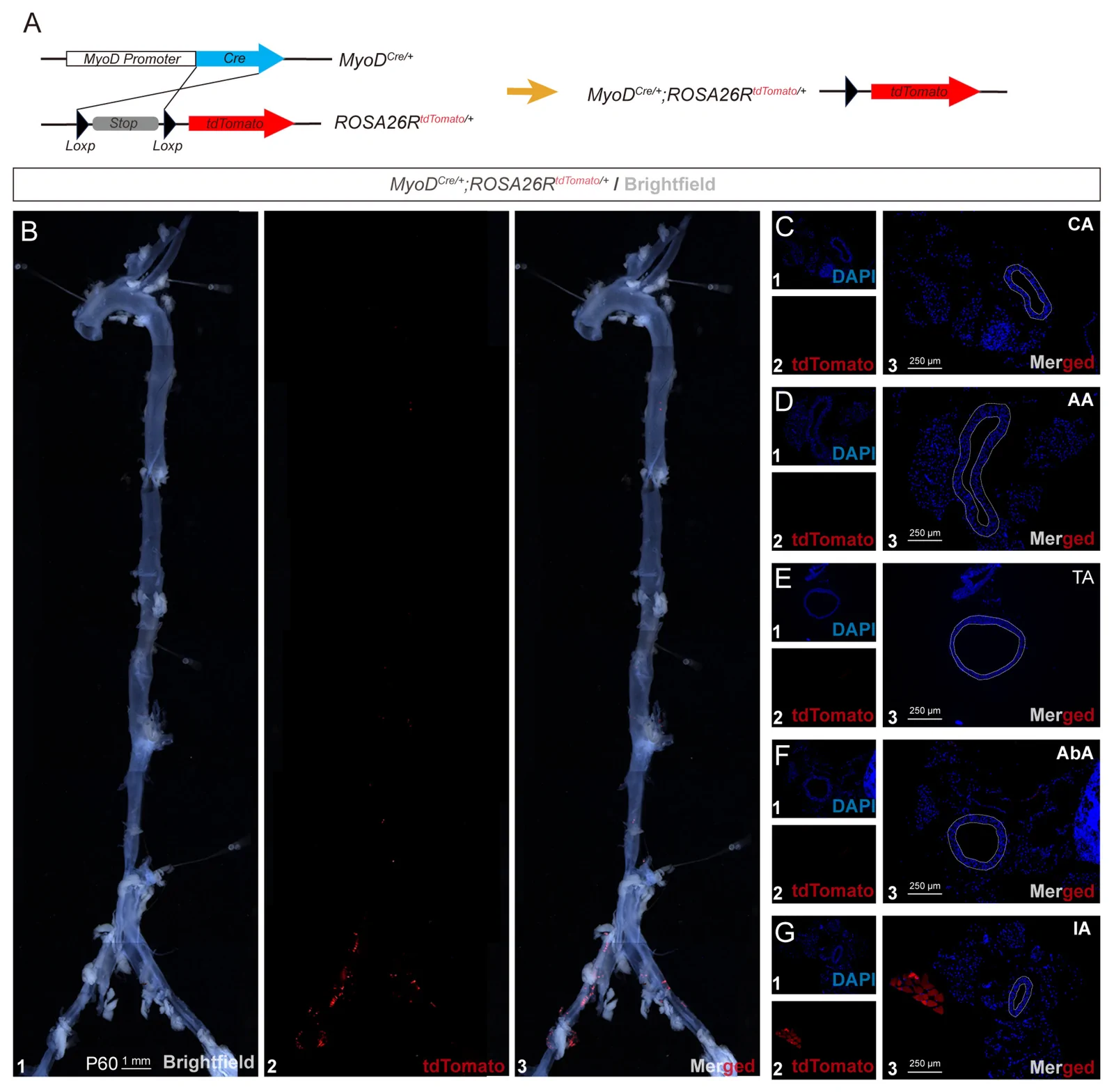
Genetic lineage tracing demonstrates the absence of *MyoD*-lineage cell contribution to large artery walls. (**A**) Schematic representation of the genetic strategy for tracking *MyoD*-derived cells using *MyoD^Cre/+^*; *ROSA26R^tdTomato/+^* mice. (**B**) Representative whole-mount brightfield (1), tdTomato fluorescence (2), and merged (3) images of the isolated murine aortic tree and major branches. Scale bars, 1 mm. (**C**–**G**) Representative fluorescence cross-section images of the carotid artery (CA, (**C**)), aortic arch (AA, (**D**)), thoracic aorta (TA, (**E**)), abdominal aorta (AbA, (**F**)), and iliac artery (IA, (**G**)). Panels show DAPI (blue, 1), tdTomato (red, 2), and merged images (3). Note the strong tdTomato signal in the adjacent skeletal muscle surrounding the IA in (**G2**,**G3**), while the arterial walls across all segments remain negative. Scale bars: 250 µm.

**Figure 3:**
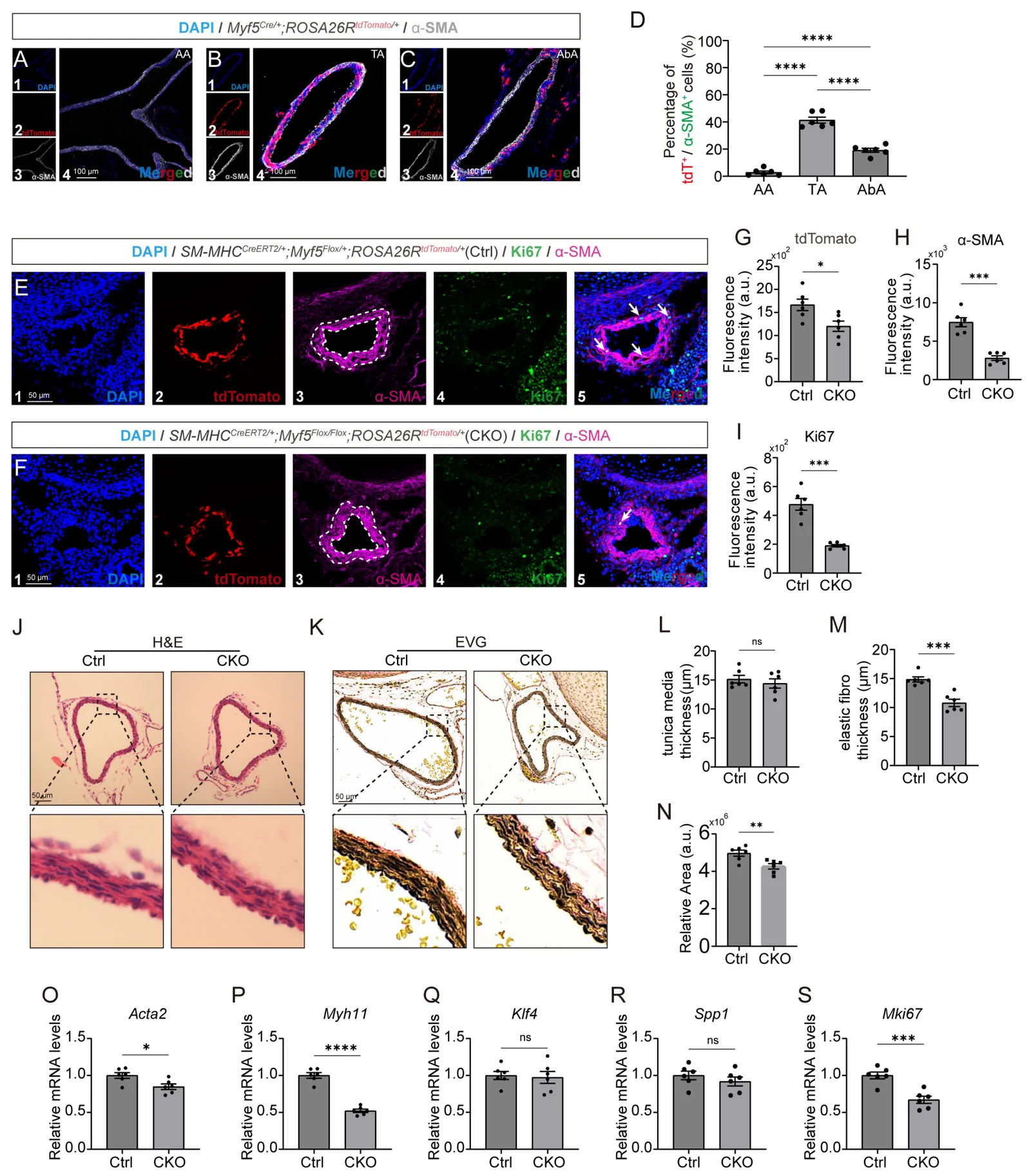
Contribution of the *Myf5 lineage* to SMA^+^ cells in aorta vessels at embryonic day 14.5 (E14.5), and the effects of *SM-MHC*-CreER-driven *Myf5* conditional knockout (CKO). (**A**–**C**) Immunofluorescence co-localization of the Myf5 lineage tracer (tdTomato, red) and α-SMA (white) in cross-sections of the aortic arch (AA) (**A**), thoracic aorta (TA) (**B**), and abdominal aorta (AbA) (**C**) from *Myf5^Cre/+^*; *ROSA26R^tdTomato/+^* embryos at E14.5. Nuclei were counterstained with DAPI (blue). Scale bars: 100 µm. Individual panels show separate channels for DAPI (1), tdTomato (2), α-SMA (3), and the merged image (4). (**D**) Quantitative analysis of the percentage of tdT^+^/α-SMA^+^ cells relative to the total α-SMA^+^ area in the AA, TA, and AbA. The contribution of *Myf5 lineage* cells in the TA was significantly higher than that in the AA and AbA, whereas the contribution in the AA was negligible. (**E**,**F**) Representative immunofluorescence images of the thoracic aorta in Ctrl (**E**) and CKO (**F**) mice showing the expression of DAPI (blue), tdTomato (red), α-SMA (magenta), and Ki67 (green). Scale bars, 50 µm. Individual panels show separate channels for DAPI (1), tdTomato (2), α-SMA (3), Ki67 (4), and the merged image (5). White dashed lines delineate the tunica media of the vessels. White arrows in the merged panels ((**E**,**F**)—5) indicate Ki67-positive cells. (**G**–**I**) Quantification of fluorescence intensity (in arbitrary units, a.u.) for tdTomato (**G**), α-SMA (**H**), and Ki67 (**I**) in Ctrl and CKO mice. (**J**,**K**) Representative hematoxylin and eosin (H&E) staining (**J**) and Elastic van Gieson (EVG) staining (**K**) of thoracic aorta cross-sections from Ctrl and CKO mice at E14.5. (**L**–**N**) Quantification of tunica media thickness (µm) (**L**), elastic fiber thickness (µm) (**M**), and relative elastic fiber area (a.u.) (**N**). Tunica media thickness showed no significant difference, whereas elastic fiber thickness and area were significantly reduced in CKO mice. (**O**–**S**) RT-qPCR validation of smooth muscle differentiation markers (*Acta2*, *Myh11*), synthetic markers (*Klf4*, *Spp1*), and proliferation marker (*Mki67*) in thoracic aortic tissue from Ctrl and CKO mice. Expression levels were normalized to β-actin (*Actb*). Scale bars: 50 µm. n = 6 per group. Data are presented as means ± SEM. * *p* < 0.05, ** *p* < 0.01, *** *p* < 0.001, **** *p* < 0.0001. ns = not significant. One-way ANOVA was used for (**D**). Student’s *t*-test was used for (**G**–**I**,**L**–**N**,**O**–**S**).

**Figure 4:**
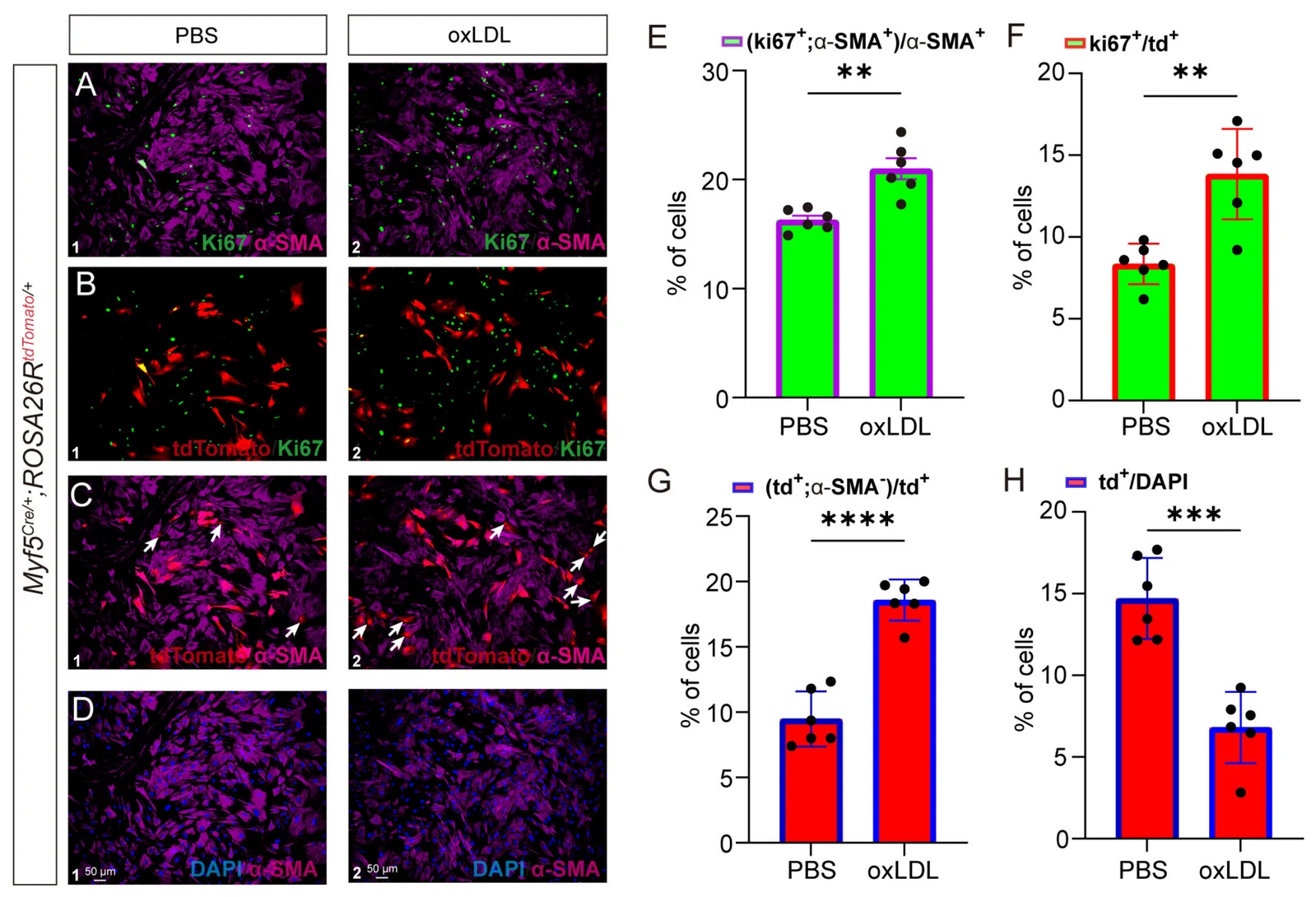
Effects of oxLDL on the proliferation and phenotypic switching of cultured *Myf5 lineage* VSMCs in vitro. (**A**–**D**) Representative immunofluorescence images of cultured primary cells treated with PBS or oxLDL, stained for: (**A**) Ki67 (green) and α-SMA (magenta); (**B**) tdTomato (red) and Ki67 (green); (**C**) tdTomato (red) and α-SMA (magenta), White arrows indicate *Myf5 lineage* cells that have lost SMA expression; and (**D**) DAPI (blue) and α-SMA (magenta). Scale bars: 50 µm. (**E**–**H**) Statistical quantification of altered cellular profiles between PBS and oxLDL treatment groups (n = 6): (**E**) percentage of (Ki67^+^/α-SMA^+^)/α-SMA^+^ cells; (**F**) percentage of Ki67^+^/td^+^ cells; (**G**) percentage of (td^+^/α-SMA^−^)/td^+^ cells; and (**H**) percentage of td^+^/DAPI cells. n = 6 per group. Data are presented as means ± SEM. ** *p* < 0.05, *** *p* < 0.001, **** *p* < 0.0001. Student’s *t*-test was used for (**E**–**H**).

**Figure 5:**
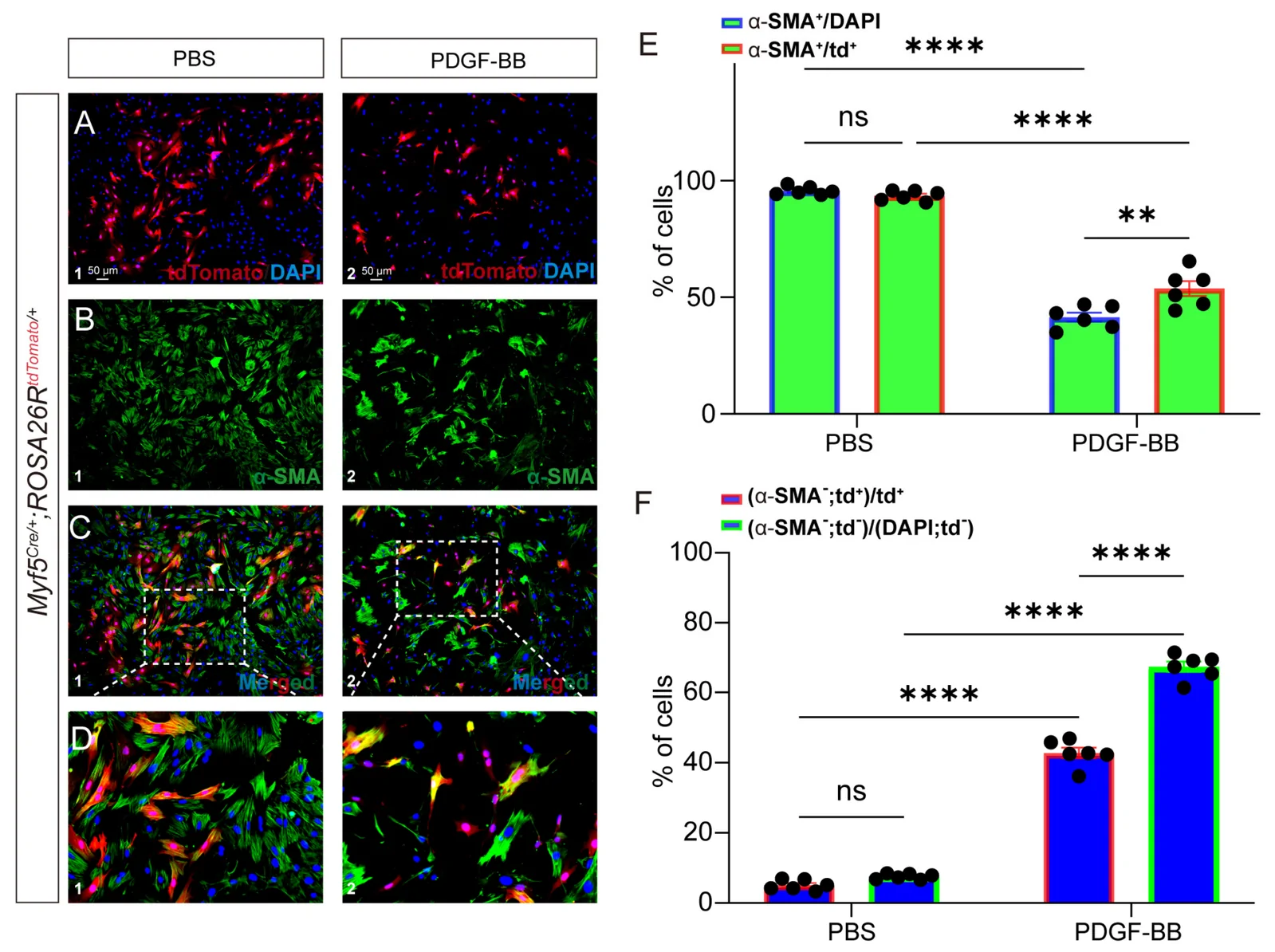
*Myf5 lineage* VSMCs display enhanced phenotypic stability against PDGF-BB-induced dedifferentiation in vitro. (**A**–**D**) Representative immunofluorescence images of primary vascular smooth muscle cells (VSMCs) from *Myf5^Cre/+^*; *ROSA26R^tdTomato/+^* mice treated with PBS or PDGF-BB. (**A**) Merged images of *Myf5 lineage* tracing (tdTomato, red) and DAPI (blue) nuclei counterstaining (**A1**,**A2**). Scale bars: 50 µm. (**B**) Immunostaining for the smooth muscle marker α-SMA (green) (**B1**,**B2**). (**C**)Triple-merged images of tdTomato, α-SMA, and DAPI (**C1**,**C2**), showing morphological alterations and downregulation of α-SMA expression in *Myf5 lineage* cells upon PDGF-BB stimulation. (**D**) Magnified views of the white dashed boxed areas in panel (**C**) and panel (**D**)—1–2, clearly illustrating the distinct phenotypic details of *Myf5 lineage*-derived (red/yellow) and non-lineage (green) VSMCs between the two groups. Scale bars: 50 µm. (**E**) Statistical analysis of the percentage of α-SMA^+^ cells relative to total cells (α-SMA^+^/DAPI^+^) and specifically within the *Myf5 lineage* population (α-SMA^+^/td^+^). (**F**) Quantitative analysis comparing the proportion of dedifferentiated (α-SMA^−^) cells within the *Myf5* lineage (α-SMA^−^;td^+^)/td^+^ versus the non-*Myf5* lineage population (α-SMA^−^;td^−^)/(DAPI^+^;td^−^). n = 6 per group. Data are presented as means ± SEM. ** *p* < 0.01, **** *p* < 0.0001. ns = not significant. Two-way ANOVA was used for (**E**,**F**).

**Figure 6:**
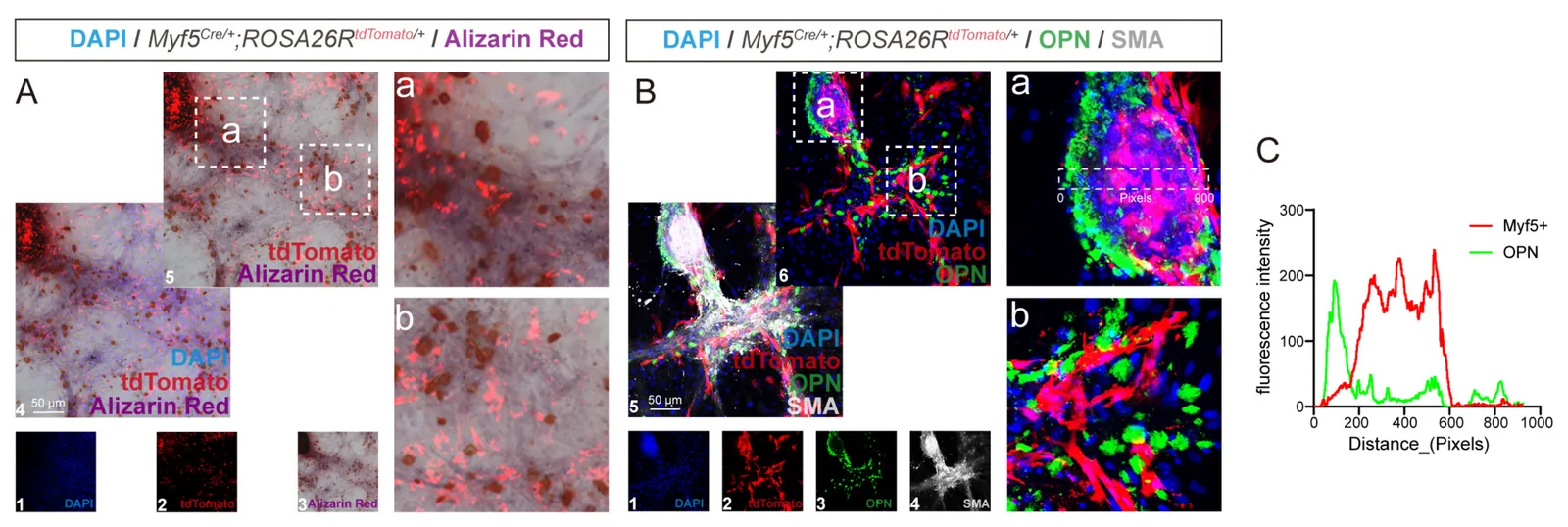
Calcified transdifferentiation capacity of *Myf5*-derived vs. non-*Myf5*-derived vascular smooth muscle cells (VSMCs) in vitro. (**A**) Representative images of Alizarin Red staining and fluorescence microscopy in *Myf5^Cre/+^*; *ROSA26R^tdTomato/+^* mouse VSMCs after calcification induction (β-glycerophosphate, ascorbic acid, and dexamethasone). 1–3: Individual fluorescence and brightfield channels showing DAPI (blue), tdTomato (*Myf5*^+^-lineage cells, red), and Alizarin Red staining (purple/calcified nodules). 4–5: Merged panels showing calcified matrix deposition relative to *Myf5*^+^ lineage tracing. a, b: High-magnification views of boxed regions highlighting spatial localization of calcium nodules relative to tdTomato-positive cells. (**B**) Immunofluorescence co-staining of osteogenic and contractile markers in calcified *Myf5^Cre/+^*; *ROSA26R^tdTomato/+^* mouse VSMCs. 1–4: Individual channels for DAPI (blue), tdTomato (red), Osteopontin (OPN, green), and α-SMA (gray). 5–6: Merged images showing distribution of osteogenic transdifferentiation markers across cell lineages. a, b: Magnified views of regions a and b. Scale bars: 50 µm. (**C**) Line scan analysis of fluorescence intensity along the dotted profile in panel ((**B**)—a).

**Figure 7:**
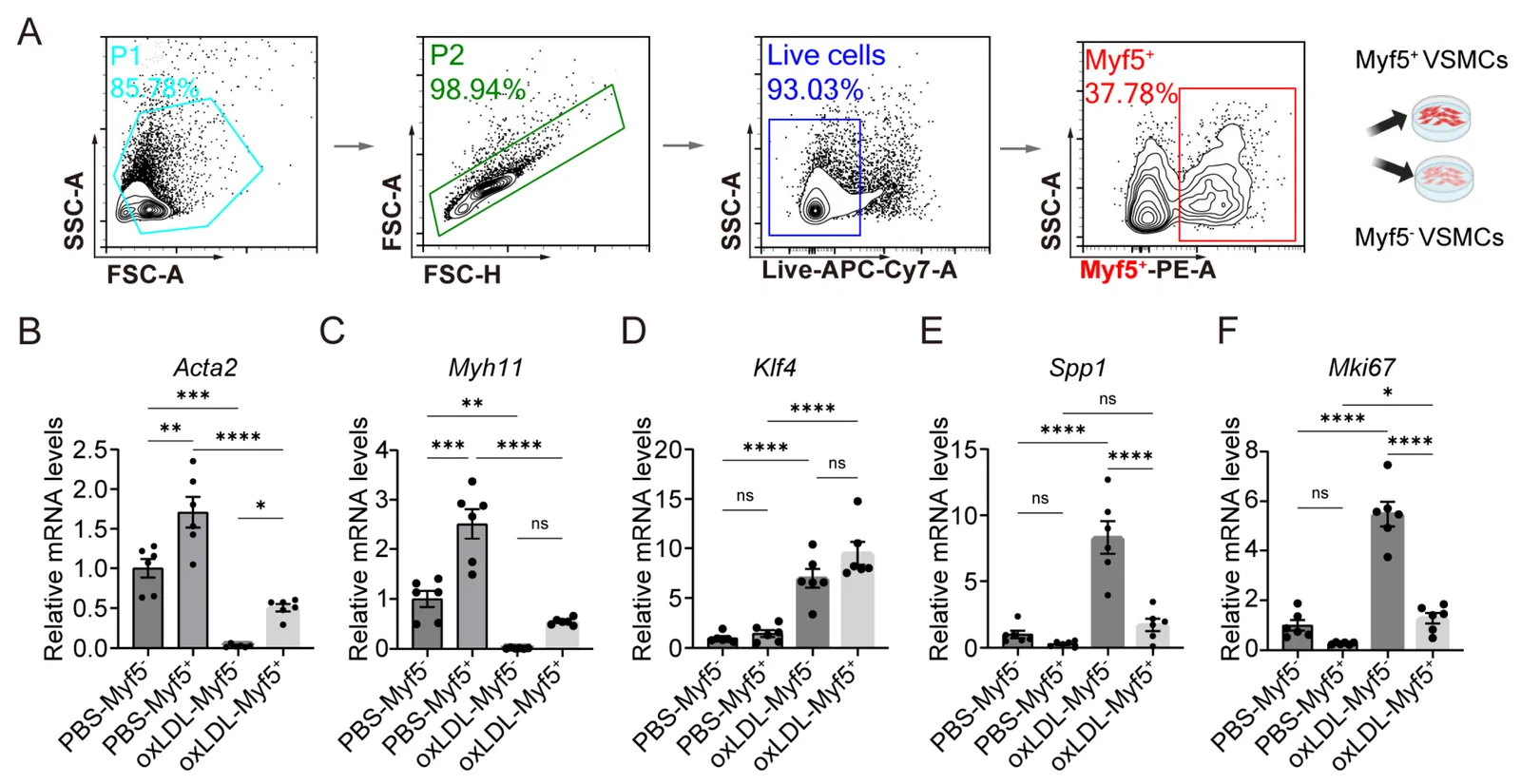
mRNA expression of phenotypic transition markers in *Myf5*^+^ and *Myf5*^−^ VSMCs under ox-LDL stimulation. (**A**) Representative FACS gating strategy for sorting primary *Myf5*^+^ and *Myf5*^−^ vascular smooth muscle cells (VSMCs). Cells were sequentially gated on cell population (P1), singlets (P2), and viable cells (Live-APC-Cy7^−^), then separated into *Myf5*^+^ (PE^+^) and *Myf5*^−^ (PE^−^) subpopulations for downstream culture and treatment. (**B**–**F**) RT-qPCR quantification of mRNA expression levels for phenotypic transition markers in sorted *Myf5*^+^ and *Myf5*^−^ VSMCs treated with PBS or ox-LDL (50 µg/mL, 48 h): contractile markers (*Acta2*, (**B**); *Myh11*, (**C**)), synthetic markers (*Klf4*, D; *Spp1*, (**E**)), and a proliferation marker (*Mki67*, (**F**)). Target gene expression was normalized to Actb (*β-actin*) and presented relative to the PBS-*Myf5*^−^ group. Data are shown as means ± SEM (n = 6 per group). * *p* < 0.05, ** *p* < 0.01, *** *p* < 0.001, **** *p* < 0.0001; ns = not significant. Two-way ANOVA was used for (**B**,**F**).

## Data Availability

The original contributions presented in this study are included in the article/Supplementary Materials. Further inquiries can be directed to the corresponding author.

## Supplementary Materials

The following supporting information can be downloaded at: https://www.mdpi.com/article/10.3390/ijms27198594/s1.
