# Supplementary material for: Myf5 Progenitors Define the Lineage Identity of Somitic Vascular Smooth Muscle Cells
Source: Int J Mol Sci. 2026 Sep 25;27(19):8594. doi: 10.3390/ijms27198594 (PMC13664892; doi:10.3390/ijms27198594)
Supplement: Supplementary file 1 [file ijms-27-08594-s001.zip › ijms-4497799-supplementary.pdf]

# Original images Figure. 1B

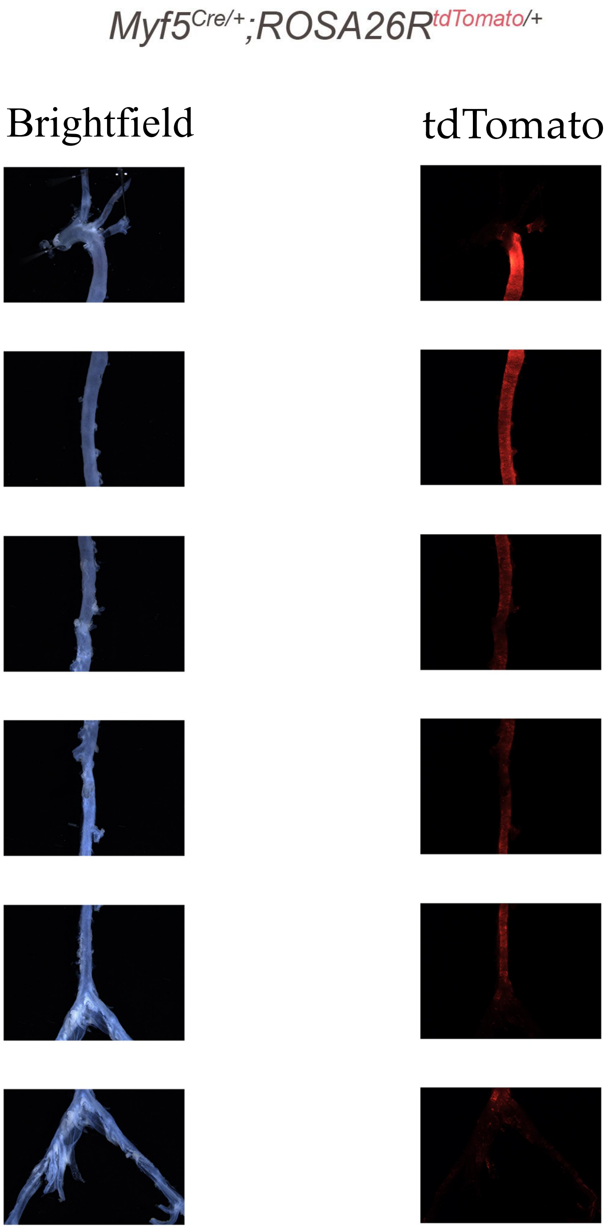

# Original images Figure. 2B

*MyoD*$^{Cre/+}$;*ROSA26R*$^{tdTomato/+}$

Brightfield    tdTomato

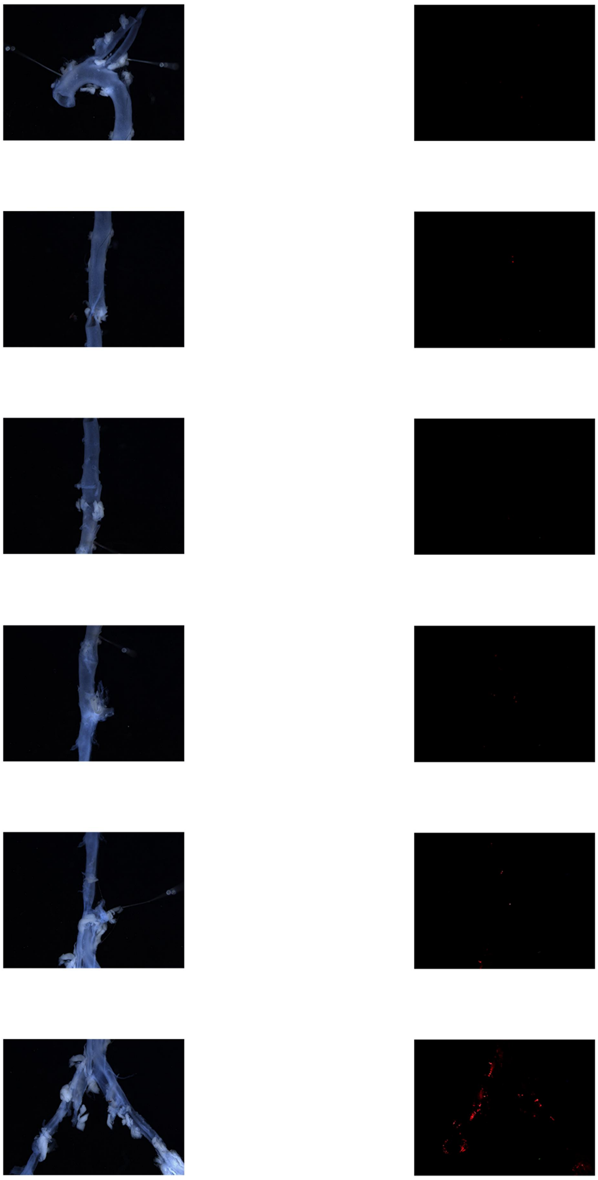

Original images Figure. 1I

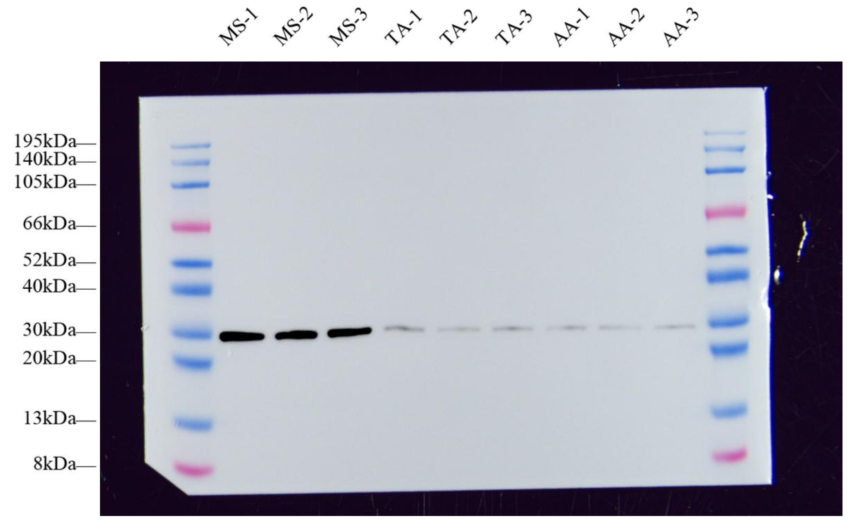


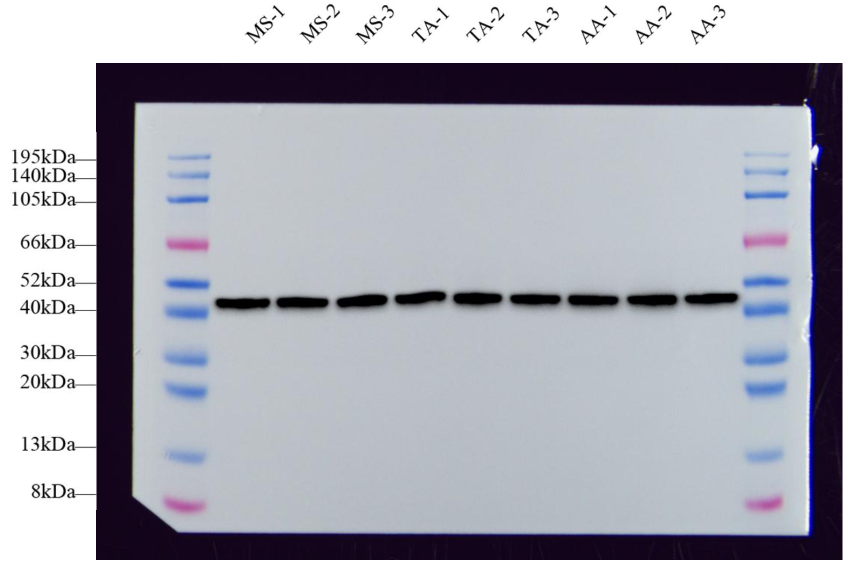

# Figure S1

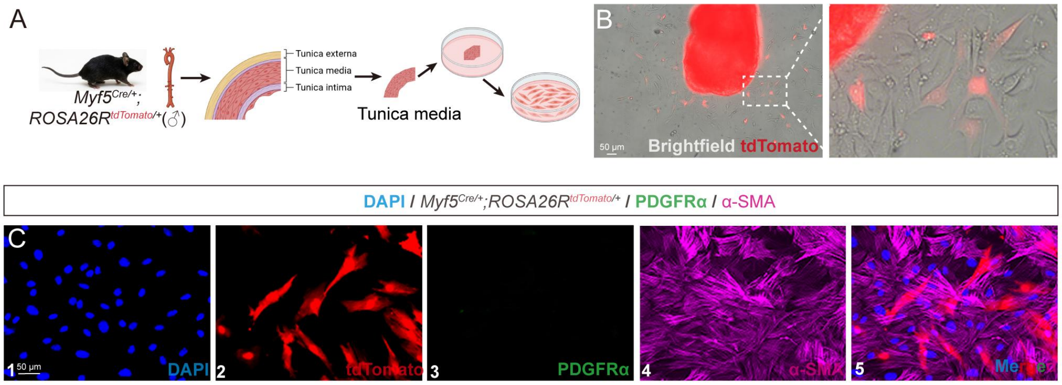


**Supplementary Figure S1.** Experimental workflow and lineage characterization of primary aortic VSMCs.

(A) Schematic diagram of the experimental workflow illustrating the isolation and harvesting method of primary VSMCs from the tunica media of *Myf5*$^{Cre/+}$; *ROSA26R*$^{tdTomato/+}$ mice. **(B)** Representative images showing primary VSMCs cultured using the explant method. The left panel shows a merged brightfield and tdTomato (red) fluorescence image, demonstrating *Myf5*-lineage-derived VSMCs migrating out of the aortic tissue explant. The right panel shows a magnified view of the dashed boxed area. Scale bar: 50 μm. **(C)** Representative immunofluorescence staining verifying cell identity and purity of isolated primary VSMCs. Cells were immunostained with DAPI (blue; nuclei, C1), tdTomato (red; Myf5-lineage marker, C2), PDGFRα (green; fibroblast marker, C3), and α-SMA (magenta; contractile VSMC marker, C4), alongside the merged image (C5). Scale bar: 50 μm.

Figure S2.

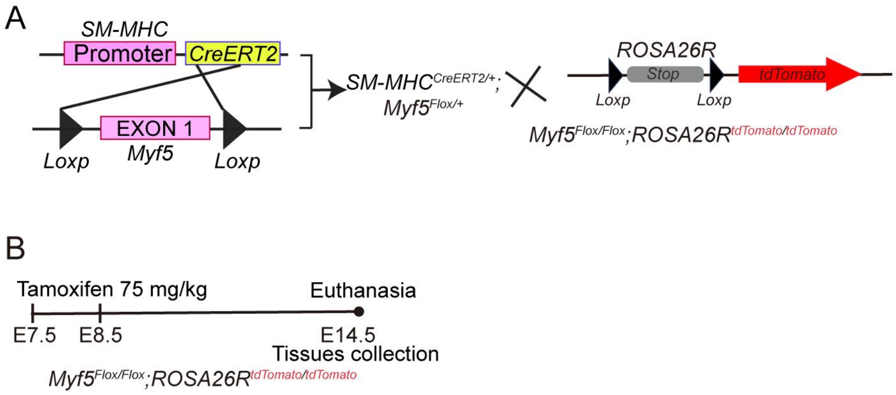


**Supplementary Figure S2.** Genetic targeting strategy and experimental induction timeline for smooth muscle-specific Myf5 conditional knockout.

**(A)** Schematic diagrams illustrating the genetic strategy used to generate *SM-MHC*$^{CreERT2/+}$; *Myf5*$^{Flox/Flox}$; *ROSA26R*$^{tdTomato/+}$ (CKO) mice and their littermate controls *SM-MHC*$^{CreERT2/+}$; *Myf5*$^{Flox/+}$; *ROSA26R*$^{tdTomato/+}$ (Ctrl). **(B)** Tamoxifen administration timeline for induction at E8.5 and tissue collection at E14.5.

Table S1

## Primer Sequence list

| Gene Symbol | Forward Primer Sequence (5' → 3') | Reverse Primer Sequence (5' → 3') |
|---|---|---|
| *Acta2* | GTCCCAGACATCAGGGAGTAA | TCGGATACTTCAGCGTCAGGA |
| *Myh11* | TCCATCGAGGTCATGGAGG | TTGTTGTTTTGGTCCTCTCCAG |
| *Klf4* | ATTATCAAGAGCTCATGCCACC | TTTCTCGCCTGTGTGAGTTC |
| *Spp1* | GGAGTTTCCAGGTTTCTGATG | GCTCAAGTCTGTGTGTTTCC |
| *Mki67* | ATGGCACCACAGCCATTATG | TTCCTCAACAGTCACTGAGTC |
| *Actb* | GCTTTCCTTCTTGGGTATGG | CAGAGCAGTAATCTCCTTCTG |
